# Autonomous 0.3-Hz breathing causes slow 0.1-Hz brain–heart oscillations in humans

**DOI:** 10.1093/cvr/cvag164

**Published:** 2026-07-27

**Authors:** Gert Pfurtscheller, Maciej Kaminski, Beate Rassler, Wolfgang Klimesch, Gerhard Schwarz, Klaus Pfurtscheller, Gaetano Valenza

**Affiliations:** Institute of Neural Engineering, Graz University of Technology, Stremayergasse16/IV, 8010 Graz, Austria; Department of Biomedical Physics, Faculty of Physics, University of Warsaw, Pasteura 5, 02-093 Warszawa, Poland; Carl Ludwig Institute of Physiology, University of Leipzig, Liebigstraße 27, 04103 Leipzig, Germany; Centre of Cognitive Neuroscience, University of Salzburg, Hellbrunnerstrasse 34, 5020 Salzburg, Austria; Department of Anaesthesiology and Intensive Care Medicine, Medical University of Graz, Auenbruggerplatz 5, 8036 Graz, Austria; Paediatric Intensive Care and Burn Unit, University Children’s Hospital and Medical University of Graz, Auenbruggerplatz 34/2, 8036 Graz, Austria; Neurocardiovascular Intelligence Lab, Department of Information Engineering and Research Center E Piaggio, University of Pisa, Largo Lucio Lazzarino 1, 56122 Pisa, Italy

**Keywords:** nasal breathing, Respiration-entrained oscillations, Cardiac 0.1-Hz oscillations, MR-related anxiety, BOLD response

**Time of primary review: 23 days**

## 1. Introduction

Brain–heart interactions are characterized by prominent oscillations centred around 0.1 and 0.15 Hz,^1^ frequencies commonly associated with the baroreflex loop (Mayer waves) and central commands.^2,3^ Beyond these slow oscillations, spontaneous respiration at approximately 0.3 Hz has been shown to modulate limbic structures, particularly the amygdala and hippocampus, in patients and healthy adults.^3–5^ Importantly, these three frequencies—0.30, 0.15, and 0.10 Hz—do not appear to be independent or randomly distributed. Rather, they follow a clear modulatory relationship. A single breathing cycle at 0.30 Hz lasts approximately 3.3 s (∼18 breaths/min). Two breathing cycles therefore span 6.6 s, corresponding to 0.15 Hz (∼9 cycles/min), while three breathing cycles span 10 s, corresponding to 0.10 Hz (∼6 cycles/min). This harmonic relationship suggests that slower cardiovascular oscillations may be organized as integer multiples of the fundamental respiratory rhythm.^6^ This interpretation is consistent with the binary hierarchy model proposed by Klimesch,^7^ which defines a carrier or preferred frequency for breathing in the range of 0.25–0.39 Hz, centred around 0.32 Hz.

## 2. Methods

Twenty-three healthy participants (mean age: 23 ± 2 years; 5 females, 18 males) underwent a comprehensive functional magnetic resonance imaging (fMRI) study comprising four resting-state sessions. The anxiety during scanning was quantified using the State-Trait Anxiety Inventory. The resulting anxiety score (AS) ranged from 10 (no anxiety) to 40 (high anxiety). Approximately half of the participants exhibited low anxiety (AS = 10–15), whereas the other half showed moderate to high anxiety (AS = 20–30).

All participants provided written informed consent, and the study protocol was approved by the Ethics Committee of the University of Graz (number: GZ. 39/75/63 ex 2013/14). Our research was performed in accordance with the ethical standards laid down in the 1964 Declaration of Helsinki. Electrocardiographic (ECG, thoracic lead), respiration (chest belt), and blood-oxygenation-level–dependent (BOLD) signals from four regions of interest—the left middle frontal gyrus (MFG), left amygdala (AMY), left hippocampus (HIP), and left caudal pons (PONSc)—were recorded using a 3 T MRI scanner with a sampling interval of 871 ms. RR interval (RRI) time series were derived from the ECG signal, whereby it is challenging to obtain artefact-free signals from the thorax during scanning. From the original cohort, 13 participants were selected based on low anxiety levels (AS = 12 ± 1.7), artefact-free ECG recordings acquired during scanning, and a breathing rate of 0.28 ± 0.04 Hz (mean ± SD).

Respiration was analysed using a wave-by-wave approach,^6^ whereas RRI peak frequency was evaluated spectrally. The RRI analyses revealed a spectral peak at 0.10 ± 0.02 Hz. Importantly, BOLD signals were not low-pass filtered prior to analysis in order to preserve their full spectral content. Multivariate autoregressive models were estimated using the RRI, respiration, and four BOLD signals, and the Directed Transfer Function (DTF) based on the Granger causality principle was computed to assess directed interactions within the 0.05–0.15 Hz frequency band. Statistical significance was determined using surrogate data and a bootstrap procedure.^5^

### 2.1 Respiration-entrained 0.3-Hz oscillations

Respiration-entrained brain oscillations can be studied most directly using invasive recordings in rodents^8^ and humans,^4^ but they can also be investigated noninvasively with fMRI in humans.^9^ Tort et al.^8^ emphasized that ‘respiration-entrained brain rhythms are global and often overlooked,’ while Zelano et al.^4^ demonstrated respiration-locked oscillations at ∼0.3 Hz in the human piriform cortex and limbic system, indicating that nasal breathing can organize neural activity across widespread brain networks.

Notably, in a cohort of healthy MRI participants, an ‘avalanche-like’ BOLD response centred at ∼0.3 Hz was observed originating in the MFG and projecting to limbic structures, including the AMY and HIP, as well as to brainstem regions containing cardiac and respiratory centres.^3,5,9^ This pattern (*Figure 1A*) likely reflects combined neuronal and humoral activation, possibly involving locus coeruleus–mediated noradrenaline release together with widespread neural recruitment operating at this carrier frequency.

**Figure 1:**
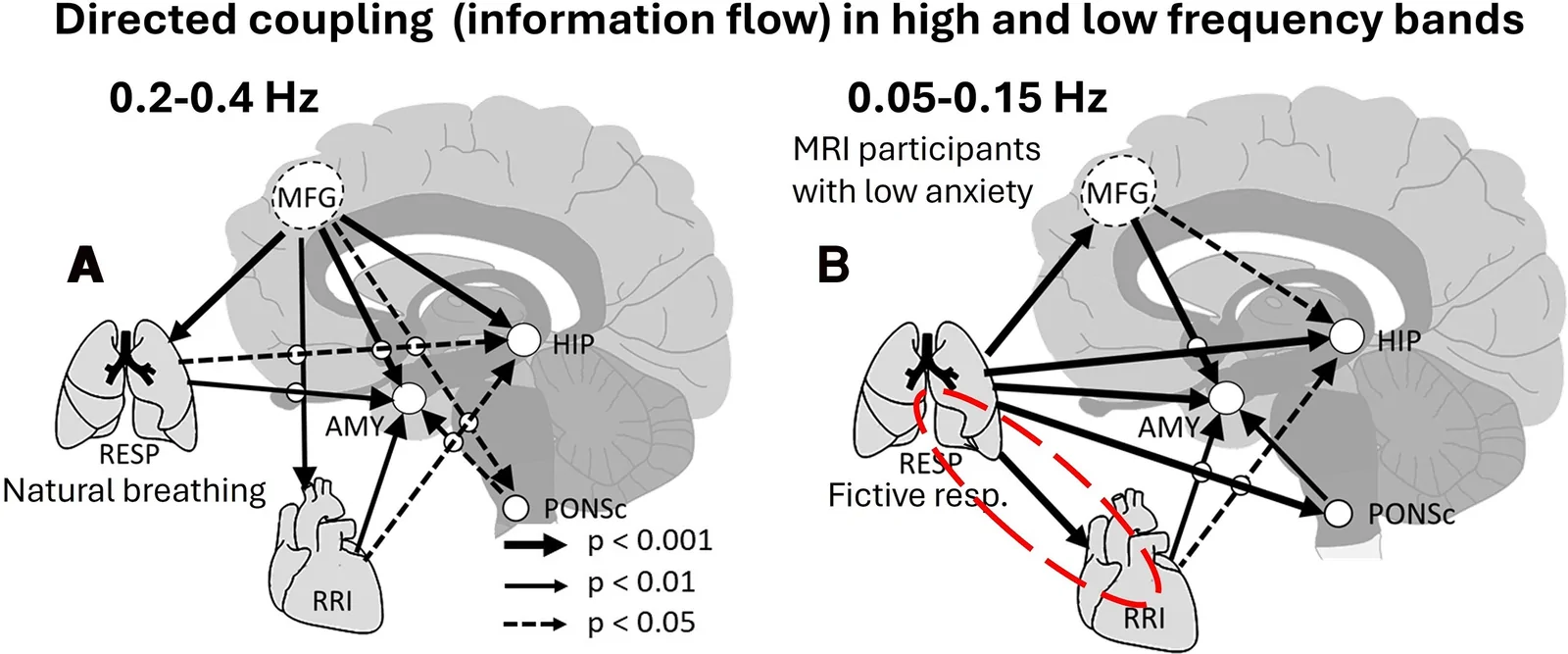
The image provides a visual summary of our key research findings from 13 healthy MRI participants. It highlights the specific brain regions studied, including cortical (middle frontal gyrus: MFG), subcortical (amygdala: AMY, hippocampus: HIP), and brainstem (pons caudal: PONSc) areas, as well as the relevant body systems (lung, RESP and heart, RRI). The image depicts the strength and direction of coupling in the frequency bands 0.2–0.4 Hz (*A*) and 0.05–0.15 Hz (*B*). Arrows indicate the significance levels of directed coupling: highly significant (*P* < 0.001, *P* < 0.01) and significant (*P* < 0.05). Surrogate data and bootstrap approach were used for statistical analyses. The characteristic directed coupling (information flow) from fictive respiration (RESP) to RRI is marked by a stippled red ellipse.

### 2.2 Fictive respiration at ∼ 0.1 Hz

The relatively small sample size required us to keep the number of analysed parameters as low as possible in order to achieve satisfactory statistical power. From the 116 BOLD signals available, four were selected. An important aspect of our DTF study is the consideration of two processing steps in different frequency bands. The first processing step is characterized by a dominant breathing rhythm near 0.3 Hz (*Figure 1A*), which is subsequently converted into respiration-like neural activity and widespread BOLD oscillations, respectively. The second processing step concerns fictive respiration at∼0.1 Hz (*Figure 1B*).

Key findings regarding the generation of respiratory rhythm in mammals originate from experimental studies based on the concept of fictive respiration. In this condition, neural activity generated by the respiratory centres in the brainstem is preserved, whereas no physical respiratory movements—and consequently no measurable airflow—occur. Experimental studies by Suzue^10^ in newborn rats demonstrated that the lower respiratory frequencies observed during fictive respiration are mainly attributable to the absence of pulmonary stretch receptor feedback and to the deafferentation of other sensory inputs. Thus, fictive respiration can be regarded as a model of pure neural respiratory coupling without mechanical feedback.

Accordingly, the term RESP denotes both natural breathing with measurable airflow in the first step and fictive respiration without airflow in the second step. Analysis within the 0.05–0.15 Hz frequency band revealed a strong directed information flow from RESP, interpreted as fictive respiration at ∼0.1 Hz, towards both BOLD and RRI signals (*Figure 1B*). This finding suggests that the RESP oscillation acts as a central driving process coupling respiratory-related neural activity with cardiovascular and haemodynamic oscillations. Simultaneously, the strong projection from RESP to BOLD signals indicates that slow respiration-related oscillations may contribute to the modulation of large-scale neural and haemodynamic activity, rather than representing merely mechanical or vascular artefacts.

The mechanism by which oscillations with a cycle duration of ∼3.3 s are transformed into slower oscillations of ∼10 s duration, corresponding to an approximate 1:3 frequency ratio, remains unresolved. One possible explanation is that widespread respiration-related BOLD oscillations at ∼0.3 Hz project to brainstem autonomic structures, where they interact with the slower arterial blood-pressure control system oscillating near 0.1 Hz, commonly associated with Mayer waves and baroreflex rhythms. Because these frequencies show an approximate 3:1 relationship, every third ∼0.3 Hz brain oscillation may coincide with a favourable phase of the cardiovascular oscillator and become selectively reinforced through phase synchronization, resonance, or nonlinear coupling mechanisms. In this way, fast limbic–cortical oscillations related to natural respiration could contribute to the emergence or stabilization of cardiac-related ∼0.1 Hz oscillations via brainstem autonomic networks. Such a mechanism would reflect a closed-loop interaction among brain activity, respiration, and cardiovascular regulation.

## 3. Conclusion

Anxiety requires rapid, largely unconscious responses mediated by the limbic system, particularly the amygdala. This emergency mechanism may begin with respiration-entrained limbic activation at ∼0.3 Hz, followed by a slower rhythmic process near 0.1 Hz, possibly orchestrated by fictive respiration and activation of brainstem autonomic networks. During this process, the RRI time course may be transformed into a continuous oscillation in the 0.05–0.15 Hz band reflecting coordinated brain–respiration–heart coupling.^9^ This fictive respiration (RESP)–heart (RRI) coupling appears to be particularly prominent during low anxiety, whereas under high anxiety conditions, the dominant direction of information flow shifts from the respiratory to the cardiac system.^3^

## Data Availability

Data available on request.
